# A Universal 3D Voxel Descriptor for Solid-State Material Informatics with Deep Convolutional Neural Networks

**DOI:** 10.1038/s41598-017-17299-w

**Published:** 2017-12-05

**Authors:** Seiji Kajita, Nobuko Ohba, Ryosuke Jinnouchi, Ryoji Asahi

**Affiliations:** Toyota Central R&D Labs., Inc., 41-1, Yokomichi, Nagakute, Aichi 480-1192 Japan

## Abstract

Material informatics (MI) is a promising approach to liberate us from the time-consuming Edisonian (trial and error) process for material discoveries, driven by machine-learning algorithms. Several descriptors, which are encoded material features to feed computers, were proposed in the last few decades. Especially to solid systems, however, their insufficient representations of three dimensionality of field quantities such as electron distributions and local potentials have critically hindered broad and practical successes of the solid-state MI. We develop a simple, generic 3D voxel descriptor that compacts any field quantities, in such a suitable way to implement convolutional neural networks (CNNs). We examine the 3D voxel descriptor encoded from the electron distribution by a regression test with 680 oxides data. The present scheme outperforms other existing descriptors in the prediction of Hartree energies that are significantly relevant to the long-wavelength distribution of the valence electrons. The results indicate that this scheme can forecast any functionals of field quantities just by learning sufficient amount of data, if there is an explicit correlation between the target properties and field quantities. This 3D descriptor opens a way to import prominent CNNs-based algorithms of supervised, semi-supervised and reinforcement learnings into the solid-state MI.

## Introduction

Discoveries of materials have been empowered by experiment, theoretical, and computational sciences. The emerging field of data science in the present day is bringing us to the fourth paradigm of the material science, in which machine learning with the experiment/simulation data automatically find a desirable material without relying on human experts^1–4^. While the great potential of this so-called materials informatics (MI) approach is becoming widely recognised, it has not yet experienced the same prevalent impact as has occurred in other scientific fields. The major difficulties of the MI arise from a descriptor, which is encoded material features through a certain protocol into digital arrays for the machine learning^5^. Design of the descriptors is one of the keys to success of the approach because the statistical models correlate the encoded representations with measured properties of materials^6–23^.

A critical obstacle to wide-spectrum applications of the MI is absence of descriptors for field quantities. A material consists of a set of electrons and nuclei. There exist several descriptors of materials that treat atoms by point representations by regarding the electrons enough localised around nuclei^13–23^. Especially in solids, however, the electrons, ionic potentials and magnetic fields tend to be delocalised over the lattice unit cell. A research group recently proposed a scheme that decomposes a target property into the local and nonlocal contributions of the field quantity; and then, these contributions are regressed instead of prediction of an entire functional of the target property itself ^24^. However, no generic descriptor for the continuous quantities in solids to directly predict the target property has been proposed yet, despite the fact that such field quantities become essential factors of solid properties such as electric conductivity, electric permittivity, and superconductivity.

Symmetry of concerning materials is also a key issue of descriptors. Because properties of materials are usually invariant with translation, rotation, and commutation of atomic labels, the descriptor itself should hold the invariance with the same operations^5,17^. It is extremely difficult to promise these invariances without dropping information of materials. In solid systems, even worse, the property is independent of choices of primitive translation vectors of the unit cell^25^. Namely, the periodicity further imposes the invariance of the unit-cell selection on solid descriptors.

In order to make a breakthrough for these problems specific to solid systems, our idea is to leverage convolutional neural networks (CNNs), which has driven a paradigm shift in computer vision and pattern recognition in terms of classifications of two- and three-dimensional objects^26,27^. The three-dimensional CNNs, which are used for human action recognitions and medical image segmentations^28–32^, are of deep models that contain trainable voxel filters and pooling operations. These characteristic layers capture global features of the three-dimensional objects, and the CNNs automatically obtain a hierarchical representation from the raw input data directly.

Here we associate the concept of the CNNs with the solid-state MI, by developing a generic voxel descriptor that represents the field quantities. The proposed voxel descriptor inherently keeps the invariances of the translation, commutation of atomic labels, and unit-cell selection; the three-dimensional CNNs learn the rotation invariance with augmented input data which are rotated from the original voxel data. This scheme allows us to predict target properties that correlate with the field quantities, without laborious efforts to design the descriptors. Here we present this scheme and comparisons with existing two descriptors in regression tests with 680-oxides data created by *ab-initio* calculations.

## Reciprocal 3D Voxel Space Descriptor with CNNs

Before showing the original descriptor for the field quantities, we briefly present two major categories in design of conventional descriptors. The most classical one is that an experienced researcher creates a set of descriptors based on relevant physical/chemical properties, such as atomic numbers, electronic negativities, band gaps, atomic or electronic densities, and core radius of pseudo electrons^6–12^. This heuristic “handcrafted descriptor” has been utilised since 1960’s at least and provided many successful results in dielectric materials^6^, alloys^8,9^, thermoelectric materials^10^, and lithium-ion conductors^11^.

The second policy is to project features of materials into a numerical vector through mathematics and theoretical physics. Unlike the handcrafted descriptors in which the choice of features is highly problem dependent, the “theoretical descriptors” do not depend on properties of users and is flexible enough to be applied to various issues. This category involves similarity of the atomic neighbour density by smooth overlap of atomic positions (SOAP)^13–15^, Coulomb matrix (CM) which consists of Coulomb potentials among constituent atoms^16–18^, representation of atomic local structure by radius symmetric functions^19–21^, Fourier and wavelet transformations of atomic destitution functions^22,23^. For the solid-state MI, the crystallography-symmetry invariances render only a few theoretical descriptor for solids, such as the alchemicaly-extended SOAP^15^ and periodically-extended CM descriptors^18^. These two descriptors, of which details are presented in the Methods section, will be used for the benchmarks later,

Let us consider a field quantity *s*(**r**) that distributes in a solid. The unit cell contains solid atoms with periodic boundary conditions regulated by primitive translation vectors **a**_**i**_, *i* = 1, 2, 3. Correspondingly, primitive translation vectors in the reciprocal space, **b**_**i**_, are defined so as to have a relation of **a**_*i*_ · **b**_*j*_ = 2 *πδ*_*ij*_.

Absolute values of the discrete Fourier’s coefficients of *s*(**r**) are expressed by the reciprocal vectors **g**, as

1 $$|s({\bf{g}})|=\frac{1}{{v}_{c}}|{\int }_{{v}_{c}}\exp (-{i}{\bf{r}}\cdot {\bf{g}})s({\bf{r}})d{\bf{r}}|,$$

where *v*_*c*_ is a volume of the unit cell. The quantity |*s*(**g**)| is translational invariance, because the operation **r** → **r** + *δ* **r** leads to

2 $$\frac{1}{{v}_{c}}|{\int }_{{v}_{c}}\exp (-i{\bf{r}}\cdot {\bf{g}})s({\bf{r}}+\delta {\bf{r}})d{\bf{r}}|=|\exp (i\delta {\bf{r}}\cdot {\bf{g}})s({\bf{g}})|=|s({\bf{g}}\mathrm{)|.}$$

Actual data of *s*(**r**) derived by numerical simulations is not continuum, but the quantity is discretised by voxels of ${\bf{r}}=({m}_{1}/{M}_{1}){{\bf{a}}}_{1}+({m}_{2}/{M}_{2}){{\bf{a}}}_{2}+({m}_{3}/{M}_{3}){{\bf{a}}}_{3}$. The reciprocal vector is also discretised as ${\bf{g}}={m^{\prime} }_{1}{{\bf{b}}}_{1}+{m^{\prime} }_{2}{{\bf{b}}}_{2}+{m^{\prime} }_{3}{{\bf{b}}}_{3}$. The integers *m*_*i*_ and ${m^{\prime} }_{i}$ are indices of the voxels, and *M*_*i*_ indicates the maximum number of the indices as $0\le {m}_{i},{m^{\prime} }_{i} < {M}_{i}$. Using the discretised field quantities *s*(**r**) ~ *s*_*r*_(*m*_1_, *m*_2_, *m*_3_) and $s({\bf{g}})\sim {s}_{g}({m^{\prime} }_{1},{m^{\prime} }_{2},{m^{\prime} }_{3})$, Eq. (1) becomes

3 $${s}_{g}({m^{\prime} }_{1},{m^{\prime} }_{2},{m^{\prime} }_{3})=\frac{{\rm{\Delta }}}{{v}_{c}}\sum _{{m}_{1},{m}_{2},{m}_{3}}\exp (-2\pi i\sum _{i}{m}_{i}{m^{\prime} }_{i}/{M}_{i})\times {s}_{r}({m}_{1},{m}_{2},{m}_{3}),$$

where ${\rm{\Delta }}$ is a volume of the voxel.

**Figure 1:**
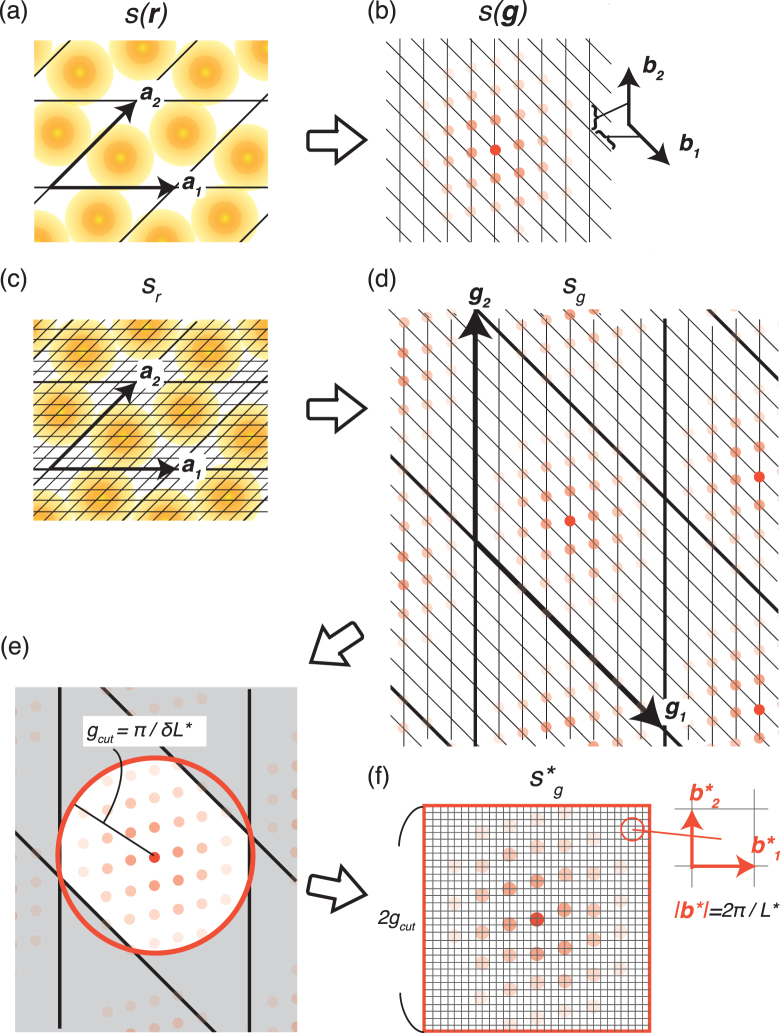
A schematic of the concept of the R3DVS descriptors. (**a**) A continuous, field quantity *s*(**r**) in a solid and (**b**) its Fourier coefficient *s*(**g**) in the reciprocal lattice space. (**c**) The discretised function *s*_*r*_ and (**d**) the corresponding Fourier’s coefficients *s*_*g*_. (**e**) The *s*_*g*_ values are hollowed out with a radius *g*_*cut*_ from **g** = 0 origin, and (**d**) the voxel values are rearranged on a new cubic meshes, which are defined by parameters δL* and L* (see the main texts).

Here we illustrate the idea of the present descriptor by focusing on influences of the discretisation on *s*(**r**) and *s*(**g**). Since *s*(**r**) is continuum and periodic (Fig. 1(a)), the Fourier transformed *s*(**g**) is discrete and non periodic (Fig. 1(b)). In the case of discrete ***r***, on the other hand, both *s*_*r*_ and *s*_*g*_ become discrete and periodic (Fig. 1(c) and (d)). The denser the voxel density in the real space, the longer the periodicity of ***g***_*i*_. In contrast to the substantial periodicity of *s*_*r*_, the periodicity of *s*_*g*_ is an artifact of the discretisation. On the basis of this key insight, it is reasonable to assume that essential features of the field quantity is not destroyed by eliminating the reciprocal periodicity. Therefore, we hollow out *s*_*g*_ with a radius *g*_*cut*_ from an origin of **g** = 0, as shown in Fig. 1(e). This extirpate operation promises the invariance of the unit-cell selection, because the selection of **a**_*i*_ only depend on the mesh shape in Fig. 1(b), but it does not change the positional configuration of the spots of *s*_*g*_. Then, the extracted *s*_*g*_ is placed in a cube on a side of 2*g*_*cut*_ as in Fig. 1(f); a set of reciprocal vectors ${{\bf{b}}}_{i}^{\ast }$ for the circumscribed cube is used to rearrange the spots of *s*_*g*_ onto the new voxels. The obtained $|{s}_{g}^{\ast }|$ is employed as a three-dimensional descriptor, which we call a reciprocal 3D voxel space (R3DVS) descriptor. The R3DVS descriptor enables us to use the three-dimensional CNNs, because it normalises any kinds of field quantities by a uniform cubic voxel, even in different solid structures.

Parameters of the present descriptor are *g*_*cut*_ = *π*/δL* and $|{{\bf{b}}}_{i}^{\ast }|=2\pi /{L}^{\ast }$. The δL* parameter defines a recaptured real-space resolution of *s*_*r*_, and the larger δL* drops rapid variances of a field quantity in real space. The other parameter L* determines a reciprocal space resolution. The smaller value of L* makes the voxel in reciprocal space coarser, causing a significant damage in the original form of *s*_*g*_ when the rearrangement shown in Fig. 1(f). Though the smaller δL* and larger L* improve accuracy of the R3DVS descriptor, they increase number of the voxels and computational costs of the CNNs. In this study, we set δL* = 0.4 Å and L* = 12.8 Å. These parameters correspond to the 32^3^ voxels in a R3DVS descriptor.

**Figure 2:**
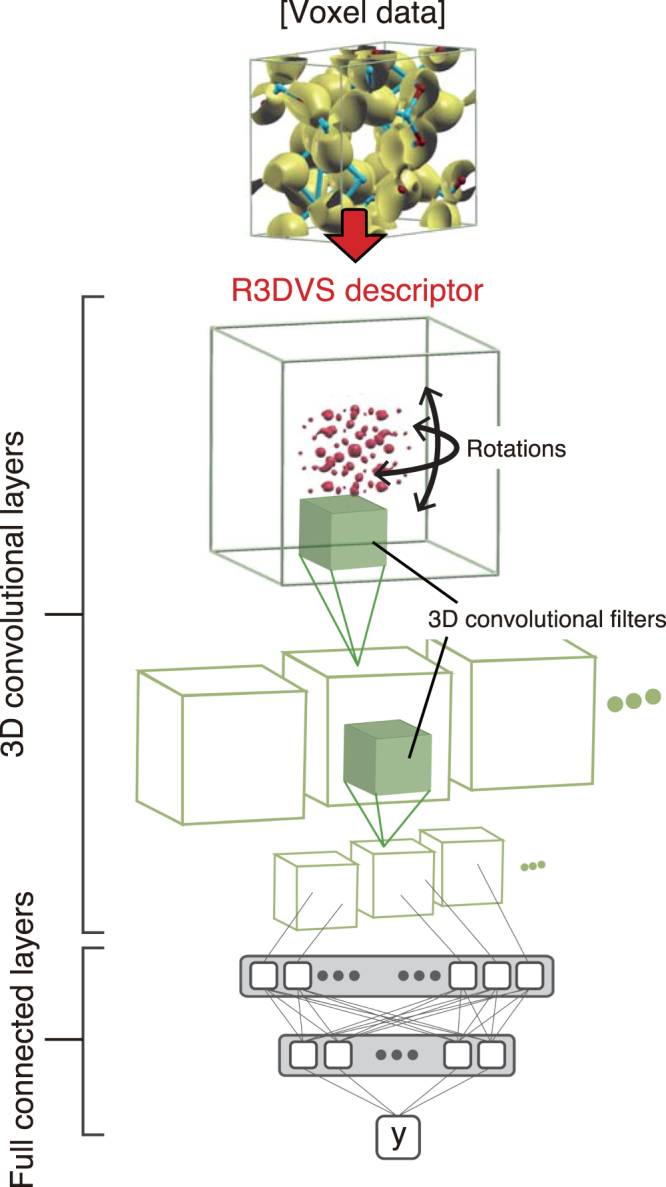
An illustration of the three-dimensional CNNs with the R3DVS descriptors. The letter “y” stands for an objective variable.

Figure 2 shows a basic architecture of the CNNs with the R3DVS input. To learn the rotation invariance, we increase the R3DVS descriptors by creating copies in which the positions of the |*s*(**g**)| spots are rotated in the cubic cell at random Euler angles on the origin **g** = 0. This type of data augmentations is commonly employed to avoid overfitting on limited input data and improve robustness of the classification abilities of the CNNs^27,29^. The rotational-augmentation R3DVS data are fed to the first convolutional layer that contains voxel filters and pooling units, and then the projected data are conveyed to the successive convolutional layers, followed by full-connected layers. The architecture of the full-connected layers depends on tasks of classification and regressions. Details of the architectures and parameters used in this study are presented in the Methods section.

## Data Preparation

Even though the practical applications should aim at predictions of properties such as non-equilibrium quantities that are difficult to be obtained by usual simulations, this study uses objective variables obtained by *ab-initio* methods for the purpose of the assessment of the present scheme.

We randomly select 680 oxides which contain less than 50 atoms in the each unit cell from the inorganic crystal structure database (ICSD; https://icsd.fiz-karlsruhe.de). Material names of the oxides is listed in the Supplementary information. The selected oxides are calculated by VASP^33^ which is a program package of electronic-state calculations based on density functional theory. Exchange-correlation functional is expressed by the Perdew-Berke-Ernzerhof type of generalised gradient approximation^34^, a plane wave basis set with a cutoff energy of 500 eV is used to expand one-electron wave function, and the projector-augmented-wave method is used to describe interactions between the valence electrons and ion cores^35^.

We adopt energy terms that constitute a total energy *E* of a unit cell as objective variables for the regression tests.

4 $$E=\sum _{i}{\varepsilon }_{i}-{E}_{H}+{\rm{\Delta }}{E}_{xc}+{E}_{I}$$

5 $${\rm{\Delta }}{E}_{xc}={E}_{xc}-\int {v}_{xc}({\bf{r}})\rho ({\bf{r}})d{\bf{r}},$$

where *ε*_*i*_, *E*_*H*_, *E*_*xc*_, *v*_*xc*_, and *E*_*I*_ indicate *i*th one-electron orbital energy, Hartree energy which is classical electron-electron electrostatic energy, exchange-correlation energy, exchange-correlation potential, and electrostatic energy of the ion cores, respectively^36^. The distribution of the electron density is denoted by *ρ*(**r**). Moreover, cohesive energy and band gap are added to the objective variables.

## Results and Discussions

### Classification to assess rotation invariance

We create the R3DVS descriptors from distributions of the valence electron density *ρ*(**r**) of the oxides. Then, the R3DVS data are augmented by copies with non-zero-angle rotations of the original R3DVS data; namely, none of the rotated replicas is identical to the original. Using the rotated replicas as the training data set, we perform classification tests if the CNNs identify the names of the target oxides when they see the original R3DVS data, in order to confirm acquisition of the rotation invariance.

**Figure 3:**
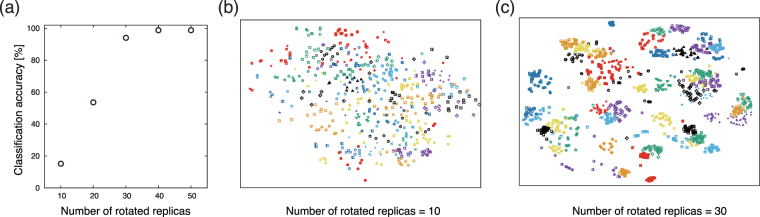
(**a**) Classification accuracies for the rotation invariance of the R3DVS descriptors. The horizontal axis indicates number of the rotated replicas that are fed into the CNNs. The vertical axis indicates classification accuracy averaged over 20 test results for randomly-chosen 50 samples of the oxides. (**b**) and (**c**) are t-SNE feature visualisations projected from the last Leaky ReLU layers embedded in the convolutional layers with 10 and 30 rotated replicas, respectively. The types of the marks indicate the 50 targets of the oxides to be classified.

The classification accuracy is evaluated by average of 20 iterations of the classification test in which 50 targets are randomly chosen from the 680 oxides. Figure 3(a) shows that the classification accuracies increases as number of the rotated replicas increases. Figure 3(b) and (c) visualise the two-dimensional features projected from the CNNs with the 10 and 30 rotated replicas by using t-SNE, respectively^37^. While the features for the 10 rotated replicas are scattered broadly, that for the 30 rotated replicas shows clusters with respect to the 50 target samples. These results indicate that the present CNNs recognises the rotation invariance with around the 30 rotated replicas; indeed, the classification accuracy at this rotation number achieves 94%. Though the present scheme does not involve the perfect rotation invariance in a mathematical form like the SOAP and CM descriptors, we consider that the 94% accuracy is practically sufficient for the purpose of prediction of a rotation-invariant objective property; thus, we use the 30 replicas for the following regression tasks.

### Regression

We generate the R3DVS descriptors from the distributions of the valence electron densities of the 680 oxide samples. These are randomly divided into the 80 oxide data as the test samples and the 600 oxide data as the training samples. Then, the training samples are augmented by the 30 rotation replicas; namely, the number of the training data amounts to 30× number of the training samples; e.g., number of the maximum training data is 30 × 600 = 18,000. The augmented training dataset is fed to the CNNs architecture to learn an objective property. After the training phase, we use the test samples to obtain the regression result by the average over the 5 answers of the CNNs trained with different random seeds, in order to reduce fluctuations of the results due to stochastically-set initial values of the CNNs architectures. The above-mentioned protocol performs 20 times with refreshing the test-sample selection; then, we calculate mean absolute errors (MAE) between the correct and averaged values of the regressions.

**Figure 4:**
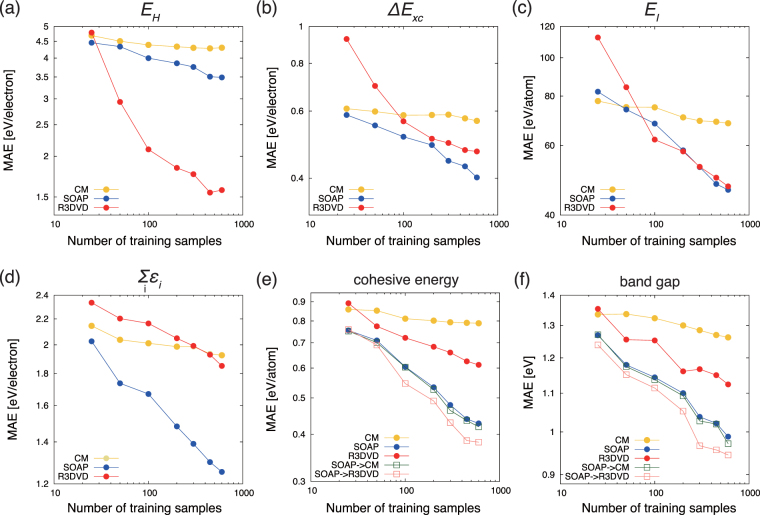
Mean absolute errors of R3DVS, SOAP and CM regressions of (**a**) Hartree energy (classical electron-electron electrostatic energy), (**b**) exchange-correlation energy term (see Eq. (5)), (**c**) electrostatic energy of ion cores, (**d**) sum of the one-electron orbital energies, (**e**) cohesive energy, and (**f**) band gap. The horizontal axis indicates number of the training samples, which do not count the rotated replicas.

Figure 4 shows the comparisons of the R3DVS, SOAP, and CM regressions with respect to the training sample size. The data of the objective variable are normalised by removing the mean and scaling to the variance, and the standardised dataset is used in the training and test phases of the regression task. The MAE results shown here are denormalised to be in the original unit. In particular, the R3DVS descriptor outperforms the others in the regressions of the Hartree energy shown in Fig. 4(a). This superiority may result from the fact that the R3DVS descriptors originate from the long-wave distributions of the valence electron densities, that are significantly relevant to target properties such as the electrostatic energy of the electrons. In Fig. 4(b) and (c), the exchange-correlation term and the electrostatic energy of the ion cores indicate almost same accuracies as those of the SOAP regressions. These performances of the R3DVS descriptors are much better than we expected in light of the fact that the exchange-correlation term could be hardly described only by the valence electrons that are source quantities of the present R3DVS descriptor, because the core electrons contribute to the target property in the form of the partial core correction^38^. Similarly, the electrostatic energy between the ion cores could be a difficult property to be guessed by the valence electron densities. The regression performance of the one-electron orbital energy (Fig. 4(d)) is worse than the others. The one-electron orbital energy includes kinetic energy and interaction energy between the electrons and ion cores. Namely, the R3DVS descriptor of the valence electrons is very insufficient information to represent these energy terms. On the other hand, we obtained the moderate performances of the R3DVS descriptors for the cases of the cohesive energy (Fig. 4(e)) and band gap (Fig. 4(f)). These results mean that the present scheme can predict functionals of field quantities, if there is an explicit correlation between the target properties and the field quantities.

The SOAP and CM descriptors treat atoms as points. They are supposed to be suitable to predict properties of ionic crystals, because the electrons much localise nearby the nuclei than those of metals do. In fact, the SOAP regression shows good, stable performances against the objective variables as shown in Fig. 4. The CM regression, on the other hand, shows the worst performances except for the regression of *E*_*I*_. Actually, the CM descriptor for solids breaks the invariance of the unit-cell selection. If one creates CM descriptors of (1 × 1 × 1) and (2 × 1 × 1) of a same solid, the two descriptors are different because of the discrepancy of numbers of the matrix elements. The deficiency of the invariance of the unit-cell selection may cause the low regression performance. Indeed, the CM regression for molecular systems, which are free from the invariance of the unit-cell selection, showed much better accuracy than that for solids^18^.

Finally, we examine to use more than one descriptor to improve the regression accuracy. The SOAP descriptor, which regards atoms as points, makes regression errors because the actual electrons spread at some extents unlike ideal point-like charges even for the localised electrons in ionic oxides. Based on this insight, we build a strategy to recover this error by the R3DVS regressions. The error Δ*y* between the correct *y* and soap-predicted *y*_SOAP_ are recorded in the training phase, and then, the Δ*y* is used for the objective variable for the R3DVS regression to create the model Δ*y*_R3DVS_. As indicated by plots with labels of SOAP → R3DVS in Fig. 4(e) and (f), the regression performance of the model of *y*_SOAP_ + Δ*y*_R3DVS_ improves. For comparison, we check the combination of the SOAP regression followed by CM regression; as shown by plots with labels of SOAP → CM, the performance improve very little. These results indicate that the concurrent use of the R3DVS and SOAP descriptors act as complementary descriptions of solids.

The R3DVS scheme has many possible extensions to improve its performance. Ways of the concurrent use of descriptors are already paved in information science, such as ensemble learning that combines plural weak classifiers and multi-modal learning that joints different neutral networks^26^. Another effective extension is use of multi channels in the three-dimensional CNNs input layer. Similar to the picture classification by CNNs with inputs in form of the three RGB channels, densities of electrons and atomic local potentials, for example, can be converted into the R3DVS descriptors as the two-channels input. Moreover, the present scheme with R3DVS descriptor also extends to the other state-of-the-arts algorithms based on CNNs, such as semi-supervised and reinforcement learnings. We hope that this study provides a new branch of confluences between MI and information science to promote further innovations in material science.

## Methods

### Architecture of CNNs

Table 1 shows detailed architecture and parameters of the CNNs in this study. The CNNs are implemented by open-source libraries of keras (https://keras.io)^39^ and tensorflow (https://www.tensorflow.org)^40^. The stride of the convolution and max pooling layers are 1 and 2, respectively. The leak parameter for negative stimulus in the leaky ReLU used in the regression is 0.3^41,42^. These CNNs are trained using the Adam optimiser^43^ with a batch size of 64. The loss functions for the classification and regression tasks are the categorical cross entropy and mean squared error, respectively.

**Table 1:** Architectures of the CNNs for the classification and regression in this study.

classification/regression		
Layer	Filter size	# of filters or elements
Convolution	3 × 3 × 3	16
Leaky ReLU	—	—
Batch normalization	—	—
Max pooling	2 × 2 × 2	—
Convolution	3 × 3 × 3	16
Leaky ReLU	—	—
Max pooling	2 × 2 × 2	—
Convolution	3 × 3 × 3	16
Leaky ReLU	—	—
Full connected	—	64/32
Leaky ReLU	—	—
Full connected	—	64/8
Leaky ReLU	—	—
Full connected	—	50/1
Soft max/Linear	—	—

### SOAP descriptor

The SOAP descriptor is a metric of similarity of two atomic environments. Suppose that a material *A* contains some elements labeled by *α*. The coordinate origin is set at a position of an *i*th atom, and the constituent atoms within a cutoff radius *r*_*cut*_ are denoted by *i*′. A density field of the environmental *α* atoms centered at the *i*th atoms is defined by

6 $${\rho }_{{A}_{i}}^{\alpha }({\bf{r}})=\sum _{i^{\prime} \in \alpha }\exp (-\frac{{({\bf{r}}-{{\bf{r}}}_{i^{\prime} })}^{2}}{2{\sigma }^{2}}),$$

where the atomic density is smoothed by a Gauss function with a standard deviation *σ*. The density fields is summed up with the elements as

7 $${\rho }_{{A}_{i}}({\bf{r}})=\sum _{\alpha \in A}{\rho }_{{A}_{i}}^{\alpha }({\bf{r}}),$$

which is called atomic-neighbour density. Then, we calculate overlaps of the atomic-neighbour densities of two materials *A* and *B*. According to rigorous definition of the atomic similarity, overlaps of different elements count zero. Nevertheless, elements on the same column in the periodic table have a chemical similarity. This alchemical insight is introduced to extend the concept of the similarity to that for different elements. Concretely, employing electronegativity *μ*, one can define the similarity of the atomic-neighbour densities by the rotational-angle integral as

8 $$k({A}_{i},{B}_{j})=\int {|\sum _{\alpha \in A,\beta \in B}{\kappa }_{\alpha ,\beta }\int {\rho }_{{A}_{i}}^{\alpha }({\bf{r}}){\rho }_{{B}_{j}}^{\beta }(\hat{R}{\bf{r}})d{\bf{r}}|}^{2}d\hat{R},$$

where ${\kappa }_{\alpha ,\beta }=\exp (-{({\mu }_{\alpha }-{\mu }_{\beta })}^{2}/2{{\rm{\Delta }}}^{2})$. The integral of the three-dimensional rotation $\hat{R}$ is numerically executable by expansions of spherical-surface harmonics basis^13^.

The matrix *k*(*A*_*i*_, *B*_*j*_) contains the complete information on the pair-wise similarity of the two systems. However, when one wants to evaluate the similarities between materials which contain different number of atoms, it is impossible to compare the matrices owing to discrepancy of numbers of the matrix elements. There are some schemes to express similarities among the plural materials; we use one of the schemes called an average structural kernel as the SOAP descriptor^15^.

9 $$\bar{K}(A,B)=K(A,B)/\sqrt{K(A,A)K(B,B)},$$

where $K(A,B)=\frac{1}{N}{\sum }_{i,j}k({A}_{i},{B}_{j})$ and *N* is number of atomic pairs of (*i*, *j*). Because oxides dataset is used in this study, the indices *i* and *j* run only on the constituent oxygen atoms.

The parameters of the SOAP are *r*_*cut*_ = 5.0 Å, *σ* = 0.5 Å, and Δ = 1, and the SOAP descriptor is used as the kernel in the ridge regression model with the regularisation parameter of 3.0. These parameters were determined by minimising the MAE shown in Supplemental Information and the references^14,15^. The regression model was implemented by the scikit-learn library (http://scikit-learn.org/stable)^44^.

### CM descriptor

The CM, of which elements are assigned to atomic pair-wise coulomb potentials, was first developed for the purpose of regressions of molecule systems^16,17^; afterwards, it was extended to that for solid systems by Ewald-sum technique^18,45^. The practical manner to generate the CM descriptor is described in the followings.

Nuclei align in a unit cell and a uniform compensation charge distributes. The element of the CM is the electrostatic energy between a pair of atoms including the periodic replicas due to the solid periodicity. Then, the matrix are diagonalised, the eigenvalues are divided by the number of the eigenvalues, and they are sorted by ascending order^17^. Finally, the eigenvalues of the smaller system is filled by zeros so as to correspond to number of the atoms in the biggest system in a dataset^16^. This numerical vector is used as the CM descriptor in this study. The regression model is the Gaussian kernel ridge with the regularisation parameter of 0.01, implemented by the scikit-learn library^44^.

## Electronic supplementary material

Supplementary Information
