# Supplementary material for: A Universal 3D Voxel Descriptor for Solid-State Material Informatics with Deep Convolutional Neural Networks
Source: Sci Rep. 2017 Dec 5;7:16991. doi: 10.1038/s41598-017-17299-w (PMC5717226; doi:10.1038/s41598-017-17299-w)
Supplement: Supplementary file 1 — Supplementary Information [file 41598_2017_17299_MOESM1_ESM.pdf]

Supplementary Information: A Universal 3D Voxel Descriptor for Solid-State Material Informatics with Deep Convolutional Neural Networks

Seiji Kajita, Nobuko Ohba, Ryosuke Jinnouchi, and Ryoji Asahi
Toyota Central R&D Labs., Inc., 41-1, Yokomichi, Nagakute, Aichi, 480-1192, Japan

I. NAMES OF 680 OXIDES

$Cr_2Mn_4Pb_4O_{18}$
$Mo_6Tl_4Se_2O_{24}$
$Ge_2Te_4O_{12}$
$Ag_{12}Sb_4O_{16}$
$Ca_4Te_4O_{16}$
$K_8S_4O_{16}$
$Rb_{12}Co_4O_8$
$K_4W_2O_8$
$Pr_4Au_2P_4O_2$
$S_6N_4O_4$
$Mo_2Zn_2O_8$
$Cs_8Ru_4O_{16}$
$Tl_8C_{16}O_{16}$
$Cr_8Tl_8O_{32}$
$Rb_8Cr_4O_{16}$
$Sc_4Te_{10}O_{26}$
$Tl_4C_6O_8$
$Rb_8C_8O_{16}$
$HgCdO_2$
$Na_4Ge_2Te_2O_{12}$
$Cu_8Se_4O_{16}$
$Ag_4C_4N_{12}O_8$
$Cs_8Ca_4V_8O_{28}$
$Na_4Y_4B_4Te_2O_{20}$
$K_6La_2Te_4O_{18}$
$Cs_{12}Cu_{20}O_{16}$
$Rb_4Mo_8Bi_4O_{32}$
$Na_4S_4O_{14}$
$Y_8Si_4O_{20}$
$Hg_4Se_4O_{12}$
HgO_2
$K_6Ga_6B_6O_{21}$
$Ba_3P_6N_8O_6$
$Mo_{10}Ag_6O_{33}$
$Cs_{12}Cr_4O_{14}$
$V_{12}Pb_2O_{22}$
$Er_4Te_{10}O_{26}$
Pb_4O_4
$Na_8Si_7O_{18}$
$Mo_2Cd_2Te_2O_{12}$
$S_{12}N_8O_{20}$
$Sr_4Pr_4O_{12}$
$K_4Li_4O_4$
$Li_2Fe_2Se_8O_{20}$
$Zn_2In_4Se_8O_{24}$
$Ba_4S_4O_{16}$
$K_3NaS_2O_8$
$K_{12}Ti_4O_{14}$
$Mg_2Bi_4O_{12}$
$CuTl_2S_2O_6$
$Fe_2S_2O_8$
$Na_4C_8O_8$
$K_4C_4N_{12}O_{24}$
$Cs_4Ag_4O_4$
$Cs_{10}Ag_2Si_6O_{18}$
$Sc_4V_4Te_8O_{32}$
$Tl_4Te_6O_{14}$
$K_8Se_4O_{16}$
$Sr_4Zn_4Se_8O_{24}$
$Na_4Ga_4Se_8O_{24}$
$PbSb_2O_6$
$Os_6S_4C_{18}O_{18}$
$K_4Ba_4P_4O_{16}$
$Fe_4Bi_4O_{12}$
$Sr_4Ni_2Se_6O_{18}$
$K_4In_4Te_8O_{24}$
$K_{12}Co_4O_8$
$Ca_2Fe_2Ge_4O_{12}$
$Na_4Y_4Se_8O_{24}$
$Pd_2Se_2O_6$
$Ga_2Te_4O_{11}$
$Cr_4Tl_8O_{16}$
$Ti_2Tl_4Ge_6O_{18}$
$Pb_8Se_8O_{24}$
$Pb_{12}O_{16}$
$Cs_4Pb_2O_6$
$Rb_2Sc_2Se_4O_{12}$
$Hg_4Cd_2S_2O_{12}$
$Sr_4Y_2Cu_6Pb_4O_{16}$
$K_6Al_2O_6$
Se_8O_{16}
$Rb_8C_4O_{12}$
$Tm_2Pb_4Sb_2O_{12}$
$La_8Si_4C_4N_8O_{16}$
$Pb_4Te_2O_{10}$
$Pb_4C_4O_{12}$
$Sm_2W_4Tl_2O_{16}$
$CsCoC_4O_4$
$Ni_4Se_4O_{12}$
$Tl_6Te_3O_{18}$
$Rb_{12}Na_4Pb_4O_{16}$
$Mo_2Mn_2Te_2O_{12}$
$Eu_4Si_8N_8O_8$
$Na_4Te_4O_{14}$
$PdS_8N_2O_{28}$
$Li_{14}P_2N_6O_3$
$Li_2N_2O_6$
$K_4Ag_4O_4$
$K_8Te_8O_{20}$
$BaAu_2S_4O_{16}$
$Rb_4Mo_6Se_2O_{24}$
$K_4Pb_2O_6$
K_4CO_4
$Sc_4Bi_4O_{12}$
SCO
$Na_2Fe_2Se_2O$
$K_{12}Ge_4O_{14}$
$K_2Y_2W_4O_{16}$
$Rb_4K_8Co_4O_{10}$
$K_8S_{12}O_{24}$
$K_2PdS_8O_{26}$
$K_8Sb_4P_4O_{24}$
$K_8Pb_4O_{12}$
$Mn_4Pb_4B_4O_{16}$
$Cs_4Li_2As_2O_8$
$Cs_4K_8Au_4O_8$
$Mo_4Bi_8O_{24}$
$K_4Ba_4As_4O_{16}$
$Na_2Cd_3P_4O_{14}$
$Cs_4Li_4S_4O_{16}$
$Hg_4Te_4O_{12}$
$Pb_6S_2O_{12}$
$Cs_4Au_4S_8O_{32}$
$Pb_8S_8C_4O_{24}$
$V_2Pb_3O_8$
$K_{12}Co_4O_{14}$
$Rb_4Y_4Se_8O_{24}$
$K_6Gd_2P_4O_{16}$
$RbTlS_2O_8$
$K_2Li_6Pb_2O_8$
$Na_8N_8O_8$
$Rb_8Ru_4O_{16}$
$K_2Li_2S_2O_8$
$Mo_4Bi_8O_{24}$
$Na_8Co_4P_8O_{28}$
$Rb_4Ba_4P_4O_{16}$
$Ba_4Zn_4Se_8O_{24}$
$Cs_4Au_4O_4$
$Hg_2C_4O_8$
$Cu_4Pb_4Te_8O_{28}$
$Li_2Ag_6O_4$
$Rb_2PdS_8O_{26}$
$Pb_8Bi_2P_2O_{16}$
$NaVS_2O_8$
$Nb_4Ag_4O_{12}$
Se_4O_8
$Li_2Dy_2W_4O_{16}$
$K_4N_{12}O_{16}$
$Na_8S_8O_{12}$
$S_{14}C_6O_2$
$Li_8W_4Te_4O_{24}$
$K_4Ag_4O_4$
$Cs_8K_8Te_4O_{20}$
$Na_{12}Au_4O_8$
$W_8Pb_8O_{32}$
$Re_4S_4O_{26}$
$Li_4Pr_4S_8O_{32}$
$Bi_4Te_2O_{12}$
$K_2CoSe_2O_6$
Se_8O_{16}
$Ag_4Te_4O_{14}$
$Na_8Te_4O_{12}$
$Ca_4S_4O_{16}$
$Pd_2Se_2O_6$
K_4CO_4
$Pb_8S_{16}O_{24}$
$W_4S_8O_{36}$
$Rb_4V_4Cu_2P_4O_{24}$
K_3NbO_8
$Li_2N_2O_6$
$La_4Ga_4Se_8O_4$
$Pb_2C_4O_8$
$CsCdN_3O_6$
$Na_8W_2N_4O_4$
$Cs_9N_9O_{27}$
$K_4N_4O_8$
$Hg_8C_8N_8O_4$
$Cs_4Ag_4O_4$
$K_8S_4N_8O_{20}$
$K_{12}P_4O_{16}$
$Gd_4Mo_2Se_6O_{24}$
$Fe_6Te_2Se_2C_{18}O_{18}$
$Sr_6Te_8O_{22}$
$Cs_2C_4O_4$
Rb_8O_{12}
$Rb_8C_8O_{24}$
$K_4Hg_4C_{12}N_{12}O_{12}$
$Cu_8Se_6O_{20}$
$Pb_4Se_8O_{20}$
$Ba_4Mo_4Se_4O_{24}$
$K_4Ga_4Se_8O_{24}$
$Cs_{12}Si_4O_{14}$
Pb_4O_6
Rb_8O_{12}
$Ba_4Se_8O_{20}$
$Cr_4Pb_4O_{16}$
$Dy_2CuTe_2S_2O_{14}$
$Ba_3Si_6N_4O_9$
$Cs_4Li_4Ge_2O_8$
$Pb_4Te_2O_{10}$
$Na_2La_2S_4O_{16}$
$Ag_4Te_4O_{12}$
$K_8Mo_4Se_4O_{24}$
$Ag_{12}C_6O_{18}$
$Cu_8Se_8O_{24}$
$Zn_8Te_8O_{24}$
$Re_4S_6C_{18}O_{16}$
$K_4La_4C_4O_{16}$
$Ba_4Mn_4Sb_4O_2$
$Hg_6Te_4Se_2O_{20}$
$Cu_4Te_4O_{12}$
$BaNb_7O_9$
$Ba_4Cu_2Tl_4O_{12}$
$K_3NaSe_2O_8$
Rb_2HgO_2
$Tl_8Te_8O_{20}$
$Pd_2N_4O_{12}$
$Pb_4Sb_4O_{14}$
$K_3NaRu_2O_8$
$Na_2Au_2S_4O_{16}$
$Pd_4Bi_2O_8$
$Li_8Mo_4Te_4O_{24}$
$Cd_8Se_8O_{24}$
$K_2MgC_2O_6$
$Rb_8Se_4O_{16}$
$RbAuSe_2O_8$
$Ba_2Fe_4Se_8O_{24}$
$Ca_4Se_4O_{12}$
$Ge_4Se_8O_{24}$
$Sr_2Te_6O_{16}$
$K_4Y_4Se_8O_{24}$
$Sr_4Fe_2Cu_2Pb_4O_{12}$
$Cs_8Mn_4O_{16}$
$Rb_{12}Cu_{20}O_{16}$
$Na_8Te_4O_{16}$
$Rb_{12}Si_4O_{14}$
$Co_4S_{12}N_{12}O_8$
Ti_8O_{16}
$Cd_4S_8O_{28}$
$K_4V_4Te_4O_{20}$
Te_4O_8
$Pb_6S_2O_{12}$
$Pd_2Se_2O_8$
$Rb_4Dy_4S_8O_{32}$
$Ho_4Te_{10}O_{26}$
$Fe_6Te_4C_{18}O_{18}$
$Cd_2Se_4O_{10}$
$K_6Lu_2P_4O_{16}$
$Ba_4Pb_4Ge_4O_{16}$
$Cs_8Cr_4O_{16}$
$Ca_2Te_6O_{16}$
$K_2MnSe_2O_6$
$Rb_4C_4O_8$
$Li_{12}Bi_4O_{16}$
$Dy_4Te_{10}O_{26}$
$Yb_4Te_{10}O_{26}$
$Cd_8Te_8O_{24}$
$Pd_2S_2O_8$
$Pb_4Se_4O_{16}$
$Cu_4Se_4O_{16}$
$La_4Cu_2Se_8O_{24}$
$Tl_4C_4O_8$
$Rb_4Li_4Ge_2O_8$
$Mg_4Te_8O_{20}$
Te_4O_{10}
$Rb_4Pb_2O_6$
$K_4Nd_4S_8O_{32}$
$Tl_6P_2O_8$
$Ce_6Si_6N_6O_{12}$
$K_6Na_2Os_4O_{18}$
$K_4Ge_4N_4O_4$
$Rb_4Au_4O_4$
$Te_6Se_2O_{16}$
$Ag_4C_4O_8$
$K_4V_4O_{12}$
$Li_8Te_8O_{20}$
Se_8O_{16}
$Rb_6S_6O_{18}$
$Sr_6Te_8O_{22}$
$Na_2La_2W_2Fe_2O_{12}$
$Li_8Bi_8O_{24}$
$K_8Pb_8O_{16}$
$Cu_4Te_4O_{16}$
$Mo_4Ag_4O_{14}$
$Pd_2Bi_4Se_8O_{24}$
$W_2Pb_4O_{10}$
$K_2Hg_3C_8N_8O_8$
$Pd_4S_8O_{28}$
$Rb_4Cu_6P_8O_{28}$
$K_2Cu_3Se_4O_{12}$
$K_2PbS_2O_8$
$Ag_4Tl_4Te_4O_{12}$
$Cr_4Bi_4O_{12}$
$Na_4Zn_4Te_6O_{18}$
$Pb_3P_2O_8$
$Rb_{16}Ge_4O_{16}$
Rb_4CO_4
$Ag_8Bi_4O_{12}$
KNO_3
$Sr_4S_4O_{16}$
$Pb_4N_8O_{24}$
$Cs_4Pb_4P_4O_{16}$
$Ba_8Cu_{12}O_{24}$
$Hg_6Se_6O_{20}$
$Y_4Te_{10}O_{26}$
$Pb_6S_2O_{12}$
$Na_4Te_8O_{18}$
$Pb_2S_2O_6$
$Cs_4V_4O_{12}$
$Tl_3N_3O_6$
$Mn_4Pb_4S_8O_{32}$
$Cs_{12}In_4O_{12}$
$Sr_4Se_8O_{20}$
S_4O_{12}
$Ba_8Tl_8O_{20}$
$Fe_6Se_4C_{18}O_{18}$
Rb_4O_{12}
$K_4Ag_4O_4$
$Tl_8Se_4O_{16}$
$Ag_6S_2C_2N_6O_{12}$
K_4O_4
$Fe_2C_2O_6$
$W_4Mg_4Pb_8O_{24}$
$Na_8S_4O_{16}$
$K_8Zr_4Si_8O_{28}$
$K_{12}Mg_2O_8$
$K_6Sm_2P_4O_{16}$
$K_2Sb_2O_6$
$RbAuS_2O_8$
$Cs_4S_4O_{16}$
$Cs_{16}Fe_4O_{14}$
$Fe_4Pb_4B_4O_{16}$
$La_6W_2Ni_2S_6O_{12}$
$K_{12}V_4S_8O_8$
$K_8Se_4S_8O_{24}$
$Pr_4Si_2C_2N_4O_8$
$Mn_8Se_8O_{24}$
$K_8Pb_2O_8$
$Nb_8Ag_8O_{24}$
$Pb_4S_2O_{10}$
$Pb_4Ge_2Se_8O_{24}$
$K_8Be_4O_8$
$Cs_8S_4O_{16}$
$Rb_{12}Na_4Ti_4O_{16}$
$Hg_9S_3O_{18}$
$K_2Na_2S_4O_{14}$
$Cs_4Mo_6Te_2O_{24}$
$Eu_5Mo_2O_{12}$
$Mn_4Se_4O_{16}$
$Na_8S_4O_{16}$
$Cu_4S_4O_{16}$
$Sr_3P_6N_8O_6$
$Mg_2S_4O_{14}$
$Ca_6Te_2O_{12}$
$Na_8Se_4O_{12}$
$Cs_4CuSi_2O_7$
$Ba_4Si_8N_8O_8$
$Gd_8S_4O_{24}$
$Na_4Se_4O_{14}$
$Pr_4Mo_2Te_8O_{28}$
$Se_2S_4N_4O_4$
$Cu_4Te_4O_{12}$
$Fe_4Tl_4C_{12}N_4O_{16}$
$V_{12}O_{20}$
$K_3NaSe_2O_8$
$K_2S_2O_8$
$Cs_{12}As_4O_{16}$
$Na_{12}Nd_4B_8O_{24}$
$Mn_2Se_4O_{10}$
$K_6Ce_2As_4O_{16}$
$Cr_4Te_8O_{22}$
$KFeS_2O_8$
Cs_4O_{12}
$Rh_{16}O_{24}$
$Na_{16}Se_4O_{20}$
$K_{12}W_4N_8O_6$
$Ba_4Mn_4Bi_4O_2$
$Nd_4Mo_2Te_8O_{28}$
$Cd_6Te_2O_{12}$
$Rb_2In_2Te_6O_{16}$
$Zn_4S_4O_{16}$
$Co_4Te_4O_{12}$
$Sc_4Se_6O_{18}$
$Ag_8Te_8O_{24}$
Te_4O_8
$Rb_4Pb_8O_{14}$
$Ce_4Te_8O_{24}$
$Tb_2CuTe_2S_2O_{14}$
$Te_2Se_2O_8$
$Ba_4Ag_{24}O_{16}$
$Ag_2S_2O_8$
$K_2Nd_2S_4O_{16}$
$Sn_2Se_4O_{12}$
$Sr_{12}B_{12}N_{12}O_{12}$
$Hg_2S_2O_8$
$K_4Ag_4P_8O_{24}$
$LiAuS_4O_{14}$
$Tl_8Se_4O_{16}$
$Ba_2Pd_2Se_4O_{12}$
$K_3NaFe_2O_8$
$Yb_6Te_3O_{18}$
$Cs_4Li_2V_2O_8$
$Zn_4Se_8O_{20}$
$Cs_3N_3O_6$
$Ba_4S_4O_{16}$
$Cd_4S_4O_{12}$
$Sr_4Pb_4Ge_4O_{16}$
Hg_3O_3
$Na_8Mo_2N_4O_4$
$Sm_4Se_6O_{18}$
$Rb_{12}Cu_4O_8$
$Na_6B_6O_{12}$
$Ba_4Sc_8Nd_8O_{28}$
$Cs_{12}Sb_4O_{16}$
$Rb_8Pb_4O_{12}$
$K_4C_2O_6$
$CsCdN_3O_6$
$K_4Ag_4C_4O_{12}$
$Cu_8Te_8O_{24}$
$Nd_4W_4Te_4O_{26}$
$NaFeSe_2O_8$
$Sr_4B_8O_{16}$
$Ba_4Se_4O_{16}$
$Li_8Te_4O_{16}$
$Rb_6Ga_2O_6$
$Na_2Tm_2S_4O_{16}$
$Cs_{16}Pb_4O_{16}$
$Fe_6S_4C_{18}O_{18}$
$Na_4Ag_4S_8O_{12}$
$K_8Co_{14}O_{28}$
$CoBi_6S_2O_{16}$
$Ag_4S_4O_{14}$
$Ag_8S_4O_{12}$
$S_{16}N_{16}O_{12}$
$Cu_2Pb_4B_4O_{12}$
$Ca_4Os_2In_2O_{12}$
$Rb_2Eu_2S_4O_{16}$
$SrFe_2Se_4O_{12}$
$Cd_8Te_8O_{28}$
$Nb_4Cu_4O_{12}$
$Na_2B_2S_4O_{16}$
$K_{24}Cu_8O_{16}$
$Pb_{12}O_{16}$
$Ba_5Cd_2Sb_4O_2$
$Co_4Ag_4P_4O_{16}$
$Zr_4S_8O_{32}$
$Rb_4V_4O_{12}$
$Sr_4Te_4O_{16}$
$Ag_4Te_8O_{22}$
$K_4C_4N_{12}O_8$
Hg_4O_8
$V_8Pb_8O_{28}$
Te_2O_4
$Zn_4Ga_{12}N_{12}O_4$
$K_2Y_2W_2Mn_2O_{12}$
$Rb_8Fe_4O_{10}$
$Sm_2CuTe_2S_2O_{14}$
$Rb_8C_4O_{12}$
Cs_3TaO_8
$Ca_4Bi_2As_2O_{12}$
$Cs_8Te_4O_{16}$
$Mo_4Pb_2Se_4O_{22}$
$Cu_4Te_8O_{20}$
$K_4P_4O_{12}$
$PbAs_2O_6$
$Pb_4Te_4O_{12}$
Li_4CO_4
$Cs_4Co_4O_8$
K_4CO_4
$Sn_4S_8O_{16}$
$Sr_4Cu_2Se_6O_{18}$
$K_8Fe_4O_{16}$
$Ca_4Mg_4Si_8O_{24}$
$Tm_2CuTe_2S_2O_{14}$
$Hg_4S_8O_{28}$
$Na_2Pr_4Ru_2O_{12}$
$Na_2Au_2S_8O_{28}$
$Rb_4V_4S_4O_{24}$
$Na_4S_4O_8$
Pb_3GeO_5
$Cd_2S_2O_8$
$Sm_2CoTe_2S_2O_{14}$
$Hg_8Te_4O_{20}$
$Tl_4Bi_4S_8O_{32}$
$V_4Tl_4Te_4O_{20}$
$Rb_{12}Tl_4O_{12}$
$K_4Se_4O_{14}$
Rb_4CO_4
$K_2Na_2S_2O_8$
$KAlS_2O_8$
$Mo_2Pb_4O_{10}$
$Hg_2S_2O_8$
$Cs_8Mo_4O_{16}$
$Ru_6Se_4C_{18}O_{18}$
$Rb_2C_4O_4$
$K_{12}Si_4O_{14}$
$K_{12}Co_4O_{10}$
$CsVS_2O_8$
$Cs_2As_6O_{16}$
$Pb_4S_4O_{16}$
$K_2Pr_2S_4O_{16}$
$Mg_4Se_8O_{20}$
Te_4O_8
$Ag_8Hg_4O_8$
$K_{12}Fe_4O_{16}$
$Cu_3Tl_2Se_6O_{18}$
$Cu_4Cd_4Se_8O_{24}$
$K_4C_2O_6$
$Au_4Se_4O_{14}$
$Na_4La_4Se_8O_{24}$
$Pt_4Pb_2O_8$
Cs_2O_2
$Ag_4Te_4N_4O_{20}$
$Ca_9Al_6O_{18}$
$Ba_4P_4Te_2O_{18}$
$Cs_4Au_4O_4$
$Ba_8Cr_4O_{16}$
$K_{12}Cd_2O_8$
$K_4Te_2O_6$
$Cs_8Co_4O_{12}$
$Na_2Nd_2W_2Mn_2O_{12}$
Bi_8O_{12}
$Pr_{12}Re_4O_{32}$
$Ba_4V_8Se_4O_{32}$
$Cs_{12}Bi_8O_{18}$
$K_6Li_2Ir_2O_8$
$Cu_2Bi_4Se_8O_{24}$
$Sr_4Fe_2Cu_2Pb_4O_{12}$
$Cs_4Al_4B_4O_{14}$
Na_4CO_4
$Pt_4Pb_8O_{16}$
$Rb_8Sn_2O_8$
$Tm_4Te_{10}O_{26}$
$Au_8S_8O_{32}$
$K_{12}Cu_{20}O_{16}$
$Pd_2Se_4O_{10}$
$Mo_2Mn_2Te_2O_{12}$
N_4O_8
$Nb_4In_4Te_8O_{32}$
$Sr_4Ag_{24}O_{16}$
$Ba_2Tb_2Mn_4O_{12}$
Hg_8O_8
$K_8Fe_4O_{10}$
$Nd_4Cu_2O_7$
$Mn_8Pb_8O_{20}$
$Rb_{12}Ge_4O_{14}$
$Y_4Si_6N_8O_6$
$K_2PbS_2O_8$
$K_{12}N_4O_{16}$
$K_4N_4O_{12}$
$Cu_8Se_8O_{24}$
$S_4C_4N_4O_{12}$
$K_4S_4O_{14}$
$Fe_6Te_2S_2C_{18}O_{18}$
$K_6Y_2B_4O_{12}$
$Eu_4V_4Se_8O_{32}$
$Sm_4Cu_2Se_8O_{24}$
$Tl_8S_4O_{16}$
Te_4O_8
$Co_3Se_2C_2O_{10}$
$Cs_6NiSi_2O_8$
$Pb_2Se_4O_{12}$
$K_4Y_4Te_8O_{24}$
$Cs_8N_8O_{24}$
Pb_4O_8
$La_6W_2Mn_2S_6O_{12}$
$Cs_8C_8O_{16}$
$Li_4S_2O_8$
Cs_4CO_4
$K_{12}Ti_4O_{14}$
$Mo_2Co_2Te_2O_{12}$
$Ag_4Hg_4Te_6O_{24}$
$Rb_2Lu_2S_4O_{16}$
$Na_{14}Fe_6O_{16}$
$Rb_4Y_4Te_8O_{24}$
$Cu_6Se_6O_{18}$
$Hg_4Se_4O_{12}$
$Ag_8Te_4O_{12}$
$Pb_4B_4Bi_4O_{16}$
$CuCO_3$
$K_6Fe_2O_6$
Se_8O_{20}
$Rb_8Mo_4Se_4O_{24}$
$Cs_{12}Fe_4O_{12}$
$Cs_2In_2Te_6O_{16}$
$K_4Na_4Ge_4O_{12}$
$Ca_4Se_6O_{16}$
$La_6W_2Co_2S_6O_{12}$
$Pb_6Ge_2O_{10}$
$Ag_8Se_4O_{12}$
$Cs_8Se_4O_{16}$
$K_6B_6O_{12}$
$Ba_6Te_8O_{22}$
$Cr_4Pb_6P_8O_{32}$
$Na_2S_2O_8$
$Cs_4S_8N_{12}O_{16}$
$Eu_4S_4O_{16}$
$Cd_6Se_6O_{20}$
$Cs_4La_4S_8O_{32}$
$Cs_4Sc_4Se_8O_{24}$
$Ba_2Te_4O_{12}$
$Cu_2Tl_8Te_2O_{12}$
$Rb_4Li_4S_4O_{16}$
$K_6S_6O_{18}$
$Pb_2Se_2O_6$
$K_4Mo_4As_4O_{24}$
$Nd_2Cr_2Te_2O_{12}$
$Ge_2Se_4O_{12}$
$Cu_4Se_4O_{12}$
Pb_6O_8
$Na_6Li_6S_6O_{24}$
$K_2Au_2S_4O_{16}$
Rb_4CO_4
$Li_2V_6Te_4O_{24}$
$K_{12}Mn_4O_{12}$
$K_8Be_6O_{10}$
$Li_8S_8O_{28}$
$Na_2Mo_3Te_3O_{16}$
$Se_4C_{16}O_8$
$K_6Ni_2O_4$
$Ag_8Cu_2Te_2O_{12}$
$Ba_2Cu_2Te_4O_{14}$
$Ce_4Se_8O_{24}$
$Co_4Bi_8S_4O_{28}$
$K_4Fe_4Se_8O_{24}$
$K_4S_4N_2O_{14}$
$Cs_4Pb_2O_6$
$Os_6S_2C_{20}O_{20}$
$Mn_6S_6O_{18}$
$Pb_3Ge_{12}O_{27}$
$Ba_2Nb_5O_9$
$K_4V_4Pb_4O_{16}$
$Gd_4Cu_2Se_8O_{24}$
$K_4Li_4C_4O_{12}$
$Hg_4Cd_2Se_2O_{12}$
$Ca_4Fe_4Se_4O_4$
Pb_8O_{12}
$Li_4B_4S_8O_{32}$
$K_6N_2O_6$
$Nd_4Mo_2Se_4O_{20}$
$Li_8PdBi_2O_{10}$
$Na_4Be_2B_4O_{10}$
$Na_4CuAs_2O_8$
$Pb_6P_8O_{26}$
$Li_4Co_6Se_8O_{24}$
$K_4Er_4S_8O_{32}$
$K_4Pr_4Se_8O_{32}$
Sb_8O_{16}
$Tl_2Ba_2Ca_3Cu_4O_{12}$
$Ca_8Te_8O_{24}$
$K_6Al_6B_6O_{21}$
$Na_4Fe_4Se_8O_{24}$
$K_4RuN_6O_{12}$
$Mn_4Se_4O_{12}$
$K_6V_2S_4O_{20}$
$Cu_4Hg_4Se_8O_{24}$
$Na_4Pd_2N_8O_{24}$
$Cs_{12}P_4O_{16}$
$Cs_2Nd_2Nb_4O_{14}$
Bi_8O_{12}
$Cu_2Se_4O_{10}$
$Pb_4S_4O_{12}$
$Os_6Se_4C_{18}O_{18}$
$Te_4S_2O_{14}$
Rb_2O_2
$K_2Eu_2Mo_4O_{16}$
$Rb_4Se_4O_{14}$
$Ag_4Pb_2P_4O_{14}$
$K_4S_2O_8$
$Mn_4Bi_4O_{12}$
$Hg_9S_3O_{18}$
$Lu_4Te_{10}O_{26}$
$Cs_4Pr_4S_8O_{32}$
$K_4S_4O_{10}$
$Rb_8S_4O_{16}$
$Cu_2N_8O_{20}$
$S_{20}N_{16}O_{12}$
$Na_4Sm_4Se_8O_{28}$
Pb_4O_4
$Fe_4Ag_4Se_8O_{24}$

II. PARAMETERS FOR THE SOAP REGRESSION

The SOAP parameters σ and Δ control the sensitivities to evaluate the similarities between two systems in terms of their atomic positions and electronegativities, respectively. Namely, the larger values make the SOAP to be less sensitive to the differences, and vice versa. Because these parameters are considered to be universal criteria in the SOAP framework, we use the same values as in the references [14, 15] in the main text: $\sigma = 0.5$ Å, and $\Delta = 1$. We now optimise the cutoff radius r_{cut} and the regularisation parameter λ in the kernel-ridge regression. The regression task for the sum of the one-electron orbital energies $\sum_i \epsilon_i$ is carried out by changing the parameters. Figure 1 shows that the optimised values in this search are $r_{cut} = 5.0$ Å, and $\lambda = 3.0$. These values are used for the regressions of all the other properties in Fig. 4 in the main text.

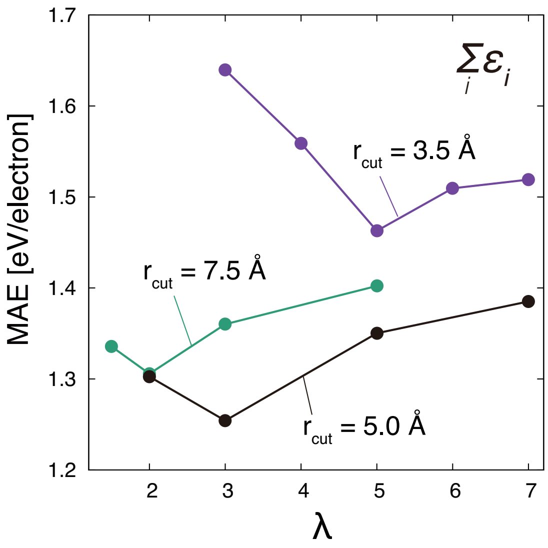

FIG. 1: Mean absolute errors of the SOAP regression for the sum of the one-electron orbital energies as functions of the cutoff radius r_{cut} and the regularisation parameter λ. The number of the training samples is 600.
